# Seizing an opportunity: increasing use of cessation services following a tobacco tax increase

**DOI:** 10.1186/s12889-015-1667-8

**Published:** 2015-04-10

**Authors:** Paula A Keller, Lija O Greenseid, Matthew Christenson, Raymond G Boyle, Barbara A Schillo

**Affiliations:** ClearWay Minnesota, 8011 34th Avenue South, Suite 400, Minneapolis, MN 55425 USA; Professional Data Analysts, Inc, St. Anthony Main, 219 Main Street SE, Suite 302, Minneapolis, MN 55414 USA

**Keywords:** Tobacco Use Cessation, Economics, Taxes, Smoking, Public Health

## Abstract

**Background:**

Tobacco tax increases are associated with increases in quitline calls and reductions in smoking prevalence. In 2013, ClearWay Minnesota^SM^ conducted a six-week media campaign promoting QUITPLAN® Services (QUITPLAN Helpline and quitplan.com) to leverage the state’s tax increase. The purpose of this study was to ascertain the association of the tax increase and media campaign on call volumes, web visits, and enrollments in QUITPLAN Services.

**Methods:**

In this observational study, call volume, web visits, enrollments, and participant characteristics were analyzed for the periods June–August 2012 and June–August 2013. Enrollment data and information about media campaigns were analyzed using multivariate regression analysis to determine the association of the tax increase on QUITPLAN Services while controlling for media.

**Results:**

There was a 160% increase in total combined calls and web visits, and an 81% increase in enrollments in QUITPLAN Services. Helpline call volumes and enrollments declined back to prior year levels approximately six weeks after the tax increase. Visits to and enrollments in quitplan.com also declined, but increased again in mid-August. The tax increase and media explained over 70% of variation in enrollments in the QUITPLAN Helpline, with media explaining 34% of the variance and the tax increase explaining an additional 36.1% of this variance. However, media explained 64% of the variance in quitplan.com enrollments, and the tax increase explained an additional 7.6% of this variance.

**Conclusions:**

Since tax increases occur infrequently, these policy changes must be fully leveraged as quickly as possible to help reduce prevalence.

## Background

Tobacco tax increases are one of the most powerful policy strategies to reduce smoking prevalence [1]. The price elasticity of demand for tobacco is well studied, with a 10% increase in the real price of cigarettes estimated to reduce smoking prevalence by 1%–2% and consumption by 2%–5% [2,3]. Tax increases result in changes in both smoking intentions and behavior. Data from the Minnesota Adult Tobacco Survey show that 65% of sampled smokers thought about quitting at the time of the 2009 federal tax increase, and 29% made a quit attempt due to the tax increase [4].

Tax increases also predict quitline service volume. Quitlines are widespread in the United States, Canada, Europe, Australia, New Zealand, and many other countries. In the U.S., state quitlines provide telephone counseling and in many cases, nicotine replacement therapy (NRT), to callers. Following the 2009 $0.62 federal cigarette tax increase, researchers examined data from 16 state quitlines and found that the tax increase was associated with a 23.5% increase in quitline utilization from December 2008 to May 2009 compared to December 2007 to May 2008 [5]. Individual states have also reported increases in call volume when their tobacco taxes increased [6–9]. Rates of change varied, ranging from more than doubling of intake calls in Montana [7] to an 1,182% increase in callers in Wisconsin [6]. Promotions and adding new services (e.g., NRT) at the time of the tax increase also positively affected call volumes [6]. However, increases in call volume may be transient. The state of Maine reported substantial increases in callers two weeks before and four weeks after a state tax increase, but then call volumes reverted to the pre-tax increase level [8].

In 2013, Minnesota enacted several large tobacco tax increases. On July 1, 2013, the sales and excise taxes on a pack of cigarettes more than doubled, increasing from $1.60 to $3.35. The tax on other tobacco products increased from 70% of wholesale price to 95% of wholesale price. Additionally, a minimum tax on smokeless tobacco went into effect on January 1, 2014; this tax was equivalent to the cigarette excise tax. This increase resulted in Minnesota’s cigarette excise tax being ranked as 7th highest in the nation [10].

ClearWay Minnesota^SM^ is an independent nonprofit organization established with three percent of Minnesota’s settlement with the tobacco industry. Since 2001, ClearWay Minnesota has funded QUITPLAN® Services. QUITPLAN Services consists of the QUITPLAN Helpline and quitplan.com. The QUITPLAN Helpline provides multi-call telephone counseling and NRT to Minnesotans who are uninsured or underinsured (i.e., no insurance coverage for telephone counseling and/or NRT). Since its inception, the QUITPLAN Helpline has transferred those with health insurance to their health plans’ quitlines to receive telephone counseling and medications through their covered benefits. In 2003, ClearWay Minnesota began offering quitplan.com, an online cessation program that is available for all Minnesotans. All QUITPLAN Services are provided at no cost to participants. ClearWay Minnesota actively promotes QUITPLAN Services through a mass media campaign that includes television, radio, billboards, bus sides, and other channels. These efforts have resulted in 71% of adult Minnesotans reporting having heard of QUITPLAN Services [11].

Several studies have shown a positive relation between paid media and quitline call volume [12–15]. Recognizing that the tax increase would motivate tobacco users to quit, ClearWay Minnesota implemented a six-week paid and earned media campaign to encourage tobacco users to enroll in QUITPLAN Services.

The aims of this study were: (1) to assess the association of a large tobacco tax increase and aggressive media campaign on call volumes, web visits, and enrollments in QUITPLAN Services; (2) to describe the association between the tax increase on enrollments in QUITPLAN Services, controlling for media; (3) to determine whether enrollee characteristics changed during the tax increase and media campaign; and (4) to understand whether enrollees endorsed the tax increase as a reason for quitting. While others have described the association of tax increases on calls and enrollments in quitline services [5–9], no one has examined these associations on both telephone counseling and stand-alone web program enrollments. Since many states, provinces and countries have quitlines, and 32 U.S. states and territories offer web-based cessation services that are either integrated with or independent from the state quitline [16], understanding the association of tax increases on both quitline and online cessation program volumes and enrollments is important to inform resource-allocation decisions. Moreover, to our knowledge, assessing the association of a tobacco tax increase on volumes and enrollments while controlling for media has not been previously reported.

## Methods

### Media campaign

To leverage the July 1, 2013 tax increase, ClearWay Minnesota undertook a six-week statewide paid and earned media campaign. The campaign began on June 24, 2013 and ended on August 4, 2013. The paid media campaign consisted of six weeks of television advertising using an advertisement that had not previously aired in Minnesota, four weeks of announcer-read radio spots promoting QUITPLAN Services, Facebook advertising, and other out-of-home advertising. Television ads were tagged approximately equally with either the QUITPLAN Services telephone number or quitplan.com. Television ad targeted rating points (TRPs; a measure of the breadth of a media campaign in a targeted audience) ranged from 125 to 150/week. The earned media campaign consisted of a press release and numerous interviews on television and radio statewide promoting QUITPLAN Services.

### Data

In this observational study, data from QUITPLAN Services vendors (National Jewish Health and Alere Wellbeing), and a media database containing information about ClearWay Minnesota’s media campaigns, were analyzed.

QUITPLAN Services data consist of administrative data on call volumes, web visits and tobacco user characteristics that are collected when a tobacco user contacts QUITPLAN Services and enrolls in either the QUITPLAN Helpline or quitplan.com. Tobacco user characteristics include demographic information (age, gender, race, ethnicity, sexual orientation, educational level, employment status, insurance status, region of the state the tobacco user resides in), clinical characteristics (pregnancy status, past year receipt of treatment for a mental health condition, whether the participant passed a medical screening to receive nicotine replacement therapy from the Helpline), tobacco use characteristics (type of tobacco used, number of cigarettes per day, time to first cigarette, e-cigarette use at intake, use of menthol cigarettes), and other data pertinent to the quitting process (intention to quit in the next 30 days, quit status at intake, quit confidence at intake, quit motivation at intake, whether the participant set a quit date during the intake process). The types of data collected for each service are shown in Tables 1 and 2.

**Table 1:** **Changes in QUITPLAN Helpline participant characteristics (N = 1828)**

***Characteristic***	***June–August 2012 (n = 696)***		***June–August 2013 (n = 1,132)***		***p-value***
	***N***	***%***	***N***	***%***	
**Gender**					0.644
Male	274	39.4	458	40.5	
Female	422	60.6	674	59.5	
**Race**					0.164
White	545	80.6	940	85.2	
Black or African American	75	11.1	95	8.6	
Asian	8	1.2	11	1.0	
Native Hawaiian or Other Pacific Islander	2	0.3	1	0.1	
American Indian or Alaska Native	16	2.4	24	2.2	
Other	7	1.0	4	0.4	
Multiple races	23	3.4	28	2.5	
**Ethnicity**					0.938
Hispanic/Latino	25	3.6	40	3.5	
Non-Hispanic	667	96.4	1089	96.5	
**Sexual orientation**					0.166
Heterosexual or Straight	644	92.7	1076	95.1	
Gay or Lesbian	24	3.5	28	2.5	
Bisexual	20	2.9	19	1.7	
Other	7	1.0	8	0.7	
**Received counseling/treatment/medication for mental health in past 12 months**					0.040
No	433	62.6	759	67.3	
Yes	259	37.4	369	32.7	
**Education**					0.932
Less than grade 9	10	1.4	15	1.3	
Grade 9 to 11, no diploma	56	8.1	101	8.9	
GED	50	7.2	77	6.8	
High school diploma	185	26.6	294	26.0	
Some college or university (includes some tech/trade school)	255	36.7	399	35.3	
College or university degree (including graduate degrees)	139	20.0	244	21.6	
**Employment**					0.236
Full-time	191	27.6	348	30.9	
Part-time	100	14.5	172	15.2	
Not working for pay/other	400	57.9	608	53.9	
**Insurance**					0.000
Uninsured	358	51.7	591	52.7	
Medicaid (includes Medical Assistance and Prepaid Medical Assistance Program)	109	15.7	93	8.3	
Medicare	137	19.8	280	25.0	
Private	42	6.1	91	8.1	
Other government (General Assistance or MinnesotaCare)	26	3.8	22	2.0	
Other – unknown	21	3.0	44	3.9	
**Region**					0.060
Minneapolis/St. Paul	280	40.3	394	34.8	
Suburban (7-county metro excluding Mpls/St. Paul)	86	12.4	148	13.1	
Rest of state	329	47.3	590	52.1	
**Pregnancy**					0.822
Yes	6	1.4	7	1.0	
Possibly	2	0.5	4	0.6	
No	414	98.1	663	98.4	
**Tobacco type**					0.144
Cigarettes (only)	644	93.7	1065	95.2	
Cigarettes and other tobacco products	20	2.9	33	2.9	
Other tobacco products (only)	23	3.3	21	1.9	
**e-Cigarette use at intake**					0.563
No	668	96.0	1080	95.4	
Yes	28	4.0	52	4.6	
**Cigarettes per day**					0.560
Less than 10	72	11.1	104	9.6	
10 to 20	431	66.5	725	66.9	
21 or more	145	22.4	255	23.5	
**Menthol cigarette user**					0.240
No	489	73.6	836	76.1	
Yes	175	26.4	262	23.9	
**Time to first cigarette**					0.908
Within 5 minutes	322	50.0	542	50.0	
6–30 minutes	213	33.1	371	34.3	
31–60 minutes	62	9.6	96	8.9	
More than 60 minutes	47	7.3	74	6.8	
**Intend to quit tobacco in next 30 days**					0.570
No	17	2.5	33	3.0	
Yes; intend to quit in next 30 days or already quit	658	97.5	1076	97.0	
**Quit status at intake**					0.054
No; currently using tobacco at intake	670	96.3	1107	97.8	
Yes; quit at intake	26	3.7	25	2.2	
**Quit confidence at intake**					0.114
Low (1–5)	153	29.1	235	25.9	
Medium (6–8)	290	55.2	551	60.8	
High (9–10)	82	15.6	120	13.2	
**Participant set a quit date during intake call**					0.568
No	425	61.1	676	59.7	
Yes	271	38.9	456	40.3	
**Medical screen**					0.000
Passed screen; no MD consent needed	590	85.4	1028	91.3	
Failed screen; MD consent needed	101	14.6	98	8.7	
Average age (stddev)	44.5 (13.6)		46.0 (14.1)		0.035

**Table 2:** **Changes in** **quitplan.com** **participant characteristics (N = 1373)**

***Characteristic***	***June–August 2012 (n = 478)***		***June–August 2013 (n = 895)***		***p-value***
	***N***	***%***	***N***	***%***	
**Gender**					0.614
Male	171	35.8	308	34.4	
Female	307	64.2	587	65.6	
**Education**					0.543
Less than high school	17	3.6	31	3.6	
High school grad or GED	95	20.4	189	21.7	
Some college/trade school	155	33.3	314	36.1	
College graduate	199	42.7	337	38.7	
**Race**					0.307
White	435	94.8	793	94.1	
Black or African American	10	2.2	27	3.2	
Asian	6	1.3	12	1.4	
Native Hawaiian / Other Pacific Islander	2	0.4	0	0.0	
American Indian or Alaskan Native	6	1.3	11	1.3	
**Ethnicity**					0.427
No	457	98.5	824	97.9	
Yes	7	1.5	18	2.1	
**Insurance**					0.065
Uninsured	120	25.6	191	21.7	
Medicaid (includes Medical Assistance and Prepaid Medical Assistance Program)	19	4.1	42	4.8	
Medicare	12	2.6	43	4.9	
Private	250	53.3	468	53.2	
Other government (General Assistance or MinnesotaCare)	4	0.9	21	2.4	
Other; unknown	64	13.6	115	13.1	
**Has health insurance**					0.107
No	120	25.6	191	21.7	
Yes	349	74.4	689	78.3	
**Sexual orientation**					0.458
Heterosexual	427	93.0	795	92.3	
Homosexual	17	3.7	26	3.0	
Bisexual	10	2.2	24	2.8	
Transgender	0	0.0	5	0.6	
Other	5	1.1	11	1.3	
**Cigarettes per day**					0.240
None	108	22.6	194	21.7	
Low (<10)	73	15.3	116	13.0	
Moderate (10 to 19)	143	29.9	314	35.1	
Heavy (20+)	154	32.2	271	30.3	
**Time to first cigarette**					0.022
Within 5 minutes after waking	112	30.3	219	31.2	
6 to 30 minutes after waking	131	35.4	300	42.8	
31 to 60 minutes after waking	86	23.2	117	16.7	
>60 minutes after waking	41	11.1	65	9.3	
**Quit status at intake**					0.529
No; currently using tobacco	382	81.4	728	82.8	
Yes	87	18.6	151	17.2	
**Quit confidence at intake**					0.642
Low (1–5)	194	41.3	340	38.7	
Moderate (6–8)	189	40.2	372	42.3	
High (9–10)	87	18.5	167	19.0	
**Quit motivation at intake**					0.113
Low (1–5)	44	9.4	83	9.4	
Moderate (6–8)	222	47.2	365	41.5	
High (9–10)	204	43.4	431	49.0	
**Employment**					0.394
Full-time	297	63.5	589	67.9	
Part-time	65	13.9	107	12.3	
Unemployed/laid-off	39	8.3	58	6.7	
Not looking for work	67	14.3	113	13.0	
Average age (stddev)	35.4 (11.4)		38.2 (11.6)		0.000

The media database consists of information about all paid media campaigns and selected earned media campaigns conducted by ClearWay Minnesota. Paid media campaigns consist of television, radio, and other types of advertising; earned media campaigns consist of free publicity generated through strategies such as press releases or interviews. Data include campaign type (e.g., quitting, social norm change), ad name, ad campaign start and end dates, and the TRPs for each television and radio ad. Data are summarized weekly. Weekly data about key earned media activities (e.g., Great American Smokeout, Minnesota State Fair) are also included.

### Analyses

Call volume, web visits, enrollments in the QUITPLAN Helpline and quitplan.com, and enrollee characteristics were analyzed for the periods June–August 2012 and June–August 2013 to assess changes at the time of the tax increase and compared to the same period in the previous year. A GLM analysis was conducted to compare monthly volumes from 2012 and 2013. Changes in the number of calls/visits and enrollments, as well as chi-squares and t-tests for differences in participant characteristics, were calculated.

To understand whether people enrolling in QUITPLAN Services reported that the tax increase motivated them to quit, ClearWay Minnesota added a question to the QUITPLAN Services enrollment process in June 2013. Enrollees were asked how much the July 1st tobacco tax increase affected their decision to quit: *a lot, some, a little,* or *not at all.* The number and percent of July and August 2013 enrollees endorsing the tax increase as a motivator was calculated.

To investigate the association of the tax increase on QUITPLAN Services enrollments controlling for the concurrent media campaign, data were analyzed from one year prior to the tax increase to six months following the tax increase (July 2, 2012 through December 29, 2013). The unit of analysis was a week, Monday through Sunday, to conform to the available TRP data for the media campaigns. Since there were 78 weeks in our analyses, a limited set of predictors was analyzed to ensure at least 10 observations per predictor [17]. Two outcome variables were used: the weekly number of Helpline enrollments and the weekly number of quitplan.com enrollments. Each outcome measure was analyzed separately using multivariate regression.

Media campaigns were coded into four predictors: statewide weekly TRPs for (1) QUITPLAN Services radio ads, (2) QUITPLAN Services TV ads, (3) “Still a Problem” (social norm change) campaign TV ads, and (4) the CDC TIPS campaign. The QuitCash Challenge, a quit-and-win contest sponsored by ClearWay Minnesota, also occurred during the study time period. This contest required that participants sign up online and also encouraged them to enroll in QUITPLAN Services. Previous evaluation data suggested increased web visits and service utilization immediately before and during the contest, so a predictor was created for the contest registration period (March 1–31, 2013) and a second predictor was created for the contest period (April 1–30, 2013). These predictors were coded as the proportion of days during a week that either contest registration or the contest itself was occurring.

Linear regression analyses were run for each outcome variable. The four media predictors and the two QuitCash Challenge predictors were all forced into the model. Because the association between the tax increase and enrollments has a time limited effect and we did not know what the duration would be or if the effect would start prior to the tax increase, we looked at the 78 residuals (observed minus predicted) from the regression. The residuals were ordered from high to low and the largest positive residuals were examined to determine the weeks in which there was a noticeable change associated with the tax increase. A binary tax impact variable was then coded to indicate the contiguous weeks during which an increase in enrollments occurred. The two linear regressions were then rerun entering the media and QuitCash Challenge predictors in Block 1 and then entering the tax impact predictor in Block 2. Data were analyzed in 2014 using SPSS version 22.0.0.0. The regression models are displayed in Tables 3 and 4.

**Table 3:** **Regression model, QUITPLAN Helpline enrollments**

**Model**	**Unstandardized coefficients**		**Standardized coefficients**	**t**	**Sig.**
	**B**	**Std. error**	**Beta**		
(Constant)	43.924	2.439		18.006	.000
QUITPLAN Radio TRPs	.062	.025	.176	2.495	.015
QUITPLAN TV TRPs	.052	.027	.145	1.928	.058
Still a Problem TV TRPs	-.011	.022	-.034	-.503	.616
CDC TIPS2 GRPs	.242	.067	.272	3.596	.001
QCC contest	15.006	7.279	.149	2.062	.043
QCC registration	3.834	7.003	.042	.547	.586
Tax impact window (5 weeks)	62.190	6.759	.680	9.202	.000

TRPs = Targeted Rating Points; GRPs = Gross Rating Points; QCC = QuitCash Challenge.

**Table 4:** **Regression model,** **quitplan.com** **enrollments**

**Model**	**Unstandardized coefficients**		**Standardized coefficients**	**t**	**Sig.**
	**B**	**Std. error**	**Beta**		
(Constant)	27.428	3.094		8.866	.000
QUITPLAN Radio TRPs	.220	.032	.483	6.920	.000
QUITPLAN TV TRPs	.161	.033	.341	4.928	.000
Still a Problem TV TRPs	.007	.028	.016	.241	.810
CDC TIPS2 GRPs	.060	.085	.052	.703	.484
QCC contest period	−7.544	9.195	-.058	-.820	.415
QCC registration period	25.404	8.833	.215	2.876	.005
Tax Impact Window (2 weeks)	55.892	12.585	.304	4.441	.000

TRPs = Targeted Rating Points; GRPs = Gross Rating Points; QCC = QuitCash Challenge.

The Minnesota Department of Health Institutional Review Board (IRB) reviewed the study and determined it to be exempt (IRB #14-338).

## Results

### Changes in volumes and enrollments

**Figure 1:**
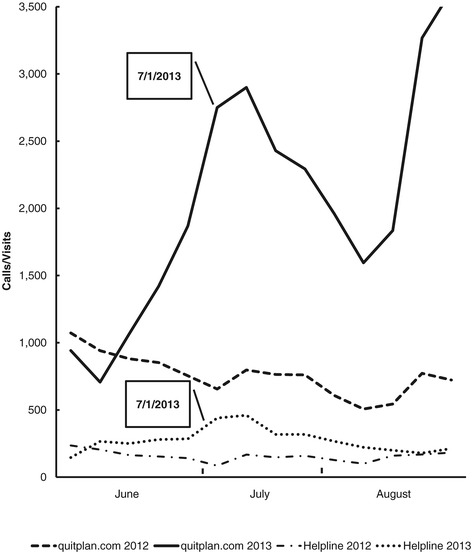
QUITPLAN services call volumes and web visits.

QUITPLAN Services saw a 160% increase in total calls and web visits combined during this period. Figure 1 illustrates changes in both call volumes and web visits for June–August 2013 compared to June–August 2012.

Calls to the QUITPLAN Helpline nearly doubled (+96%). Increases varied by month (63% in June 2013, 175% in July 2013, and 56% in August 2013), with the greatest increase occurring in the month that the tax increase was implemented. Year-over-year monthly differences in call volume were statistically significant for each month (June: p = 0.041, July: p < 0.001, August: p = 0.033). By mid-August 2013, QUITPLAN Helpline call volumes were near the same levels as mid-August 2012.

Visits to quitplan.com increased by 173% in this period. Increases of 48% in June 2013, 248% in July 2013, and 256% in August 2013 were seen. Year-over-year monthly differences in visits were statistically significant in July and August (p < 0.001 in both months); the change seen between June 2012 and June 2013 was not statistically significant (p = 0.308). A second increase in visits to quitplan.com was seen in mid-to-late August in both 2012 and 2013, although the increase was much larger in 2013 compared to 2012. Some of this secondary increase can likely be attributed to additional media activity that began in mid-August to promote quitplan.com.

**Figure 2:**
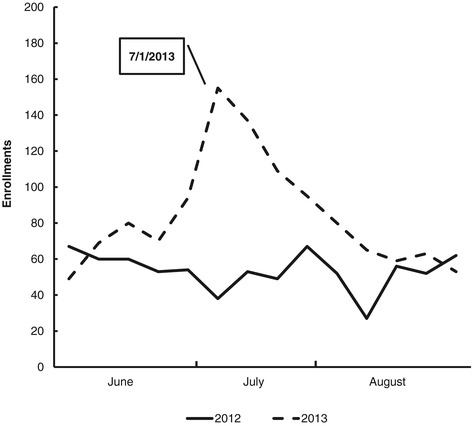
QUITPLAN helpline enrollments.

**Figure 3:**
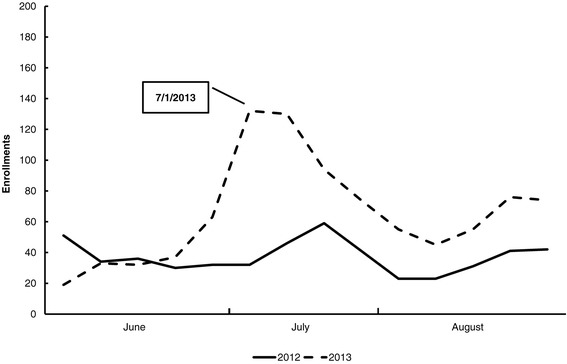
quitplan.com enrollments.

Figures 2 and 3 illustrate changes in Helpline and quitplan.com enrollments, respectively. Enrollments in QUITPLAN Services increased by 81% from June to August 2013.

QUITPLAN Helpline enrollments increased by 73% during this period (38% in June 2013, 140% in July 2013, and 43% in August 2013). Helpline enrollments mirrored the pattern observed for call volumes, returning to prior year levels by mid-August 2013 (Figure 2). The year-over-year difference in monthly enrollments was statistically significant in July (p < 0.001); the differences seen in June and August were not statistically significant (p = 0.166 and p = 0.149, respectively).

Enrollments in quitplan.com increased by 93% from June to August 2013. Increases of 25% in June 2013, 142% in July 2013, and 96% in August 2013 were seen. A second increase in enrollments was observed in mid to late August and mirrored the increase that occurred in 2012 (Figure 3). This difference was statistically significant in July (p < 0.001) and August (p < 0.009); the difference in June was not statistically significant (p = 0.937). Some of the secondary increase can likely be attributed to additional media activity that began in mid-August to promote quitplan.com.

### Association of tax increase on enrollments controlling for media

Both media and the tax increase substantially increased enrollments in the QUITPLAN Helpline. The media variables explained 34.0% of the variance in Helpline enrollments (R^2^ = 0.340, df 6, 71, p < 0.001). When the tax increase was added, 70.1% of the variance in Helpline enrollments was explained (R^2^ = 0.701, df 1, 70, p < 0.001).

A different pattern was seen for quitplan.com enrollments. The media variables explained 64% of the variance in quitplan.com enrollments (R^2^ = 0.636, df 6, 71, p < 0.001). When the tax increase was added, 71.6% of the variance was explained (R^2^ = 0.716, df 1,70, p < 0.001).

### Changes in participant characteristics

Tables 1 and 2 summarize the demographic, tobacco use and other participant characteristics analyzed in this study. Few changes in participant characteristics were observed during June–August 2013 compared to the previous year. The only significant differences (*p* ≤ 0.05) for QUITPLAN Helpline enrollees were: less likely to receive counseling or treatment for mental health conditions in the last 12 months (37.4% vs 32.4%); insurance type (less likely to have Medicaid [15.7% vs 8.3%] and more likely to have Medicare [19.8% vs 25%]); more likely to pass the medical screening to receive NRT (85.4% vs 91.3%); and older (mean age 44.5 vs 46) (Table 1). The only significant differences for quitplan.com enrollees were time to first cigarette (more reporting first cigarette between 6 and 30 minutes of waking [35.4% vs 42.8%] and fewer reporting first cigarette between 31 and 60 minutes of waking [23.2% vs 16.7%]) and older age (mean age 35.4 vs 38.2) (Table 2).

### Tax increase as motivator

The majority of tobacco users who enrolled in QUITPLAN Services in July and August 2013 endorsed the tax increase as motivating their quit attempt. Among QUITPLAN Helpline enrollees, 52.5% said that the tax increase affected their decision to quit a lot, 19.6% reported that it had some effect, 12.4% reported a little, and 15.5% reported not at all. Similar results were seen for quitplan.com enrollees, with 49.5% of enrollees stating it affected their decision to quit a lot, 26.1% reported it had some effect, 13.2% reported it had a little impact, 9.4% reported it did not affect their decision at all, and 1.8% of responses were missing.

## Discussion

Minnesota’s July 2013 cigarette tax increase, combined with an aggressive paid and earned media campaign, was associated with almost a doubling of Helpline calls and nearly a tripling of web visits compared to the same period in 2012. Substantial increases in enrollments in services were also seen. However, this increase was relatively short-lived. While Helpline call volumes and enrollments began to trend upward shortly before the tax increase, they declined to the same levels as 2012 approximately six weeks after the tax increase. This is similar to the pattern reported by Woods and Haskins [8], who observed increases in call volumes two weeks prior to Maine’s tobacco tax increase and for four weeks following the tax increase, with call volumes then returning to prior-year levels.

In this study, a second mid-August increase in quitplan.com visits and enrollments was observed. Much of this can be attributed to an annual set of promotional activities that begin in mid-August. These activities include booths at the Minnesota State Fair and extensive earned media promoting QUITPLAN Services. In 2013, two additional promotions were added: a four-week paid radio advertising campaign that was tagged with quitplan.com; and “Together We Quit”, a promotion that encouraged smokers to sign up online and quit for the month of September. Registrants were entered into a weekly drawing for a $100 gift card. The online registration site was linked to quitplan.com. In 2012, state fair activities were augmented with three weeks of television advertising that promoted quitplan.com and the QUITPLAN Helpline equally.

There is clear evidence that media campaigns as well as tax increases increase quitline call volumes [6–9,15,18]. However, in this study and in Maine, this increase was not sustained for a long period of time [8]. To take advantage of tax increases, states should aggressively promote the availability of cessation services through either paid or earned media. These data and data from other states suggest that there is a relatively narrow window of opportunity to increase demand for cessation services. The tobacco control, public health, and healthcare communities must fully leverage tax increases to help increase quit attempts and reduce prevalence. Failure to do so is a missed opportunity to help smokers attempt to quit.

The behavioral economics literature suggests that tobacco tax increases can function as a commitment device for smoking cessation [19,20]. Choi and Boyle [4] describe commitment devices as “strategies that reduce the utility of smoking to enable smokers to commit to cessation, potentially through supporting smokers to act on their intention to quit smoking”. In this paper, smokers who reported the 2009 federal tax increase as helpful for smoking cessation were more likely to make a quit attempt. The findings from the current study further support the idea that tax increases serve as commitment devices, since approximately half of QUITPLAN Services enrollees said that the state tax increase motivated their quit attempt a great deal. Additionally, data from the 2014 Minnesota Adult Tobacco Survey found that 60.8% of past-year smokers thought about quitting as a result of the 2013 price increase, and 44.2% tried to quit [21]. Recognizing the power of tax increases to spur behavior change, combined with data demonstrating that smokers use price-minimizing behaviors to quickly adapt to tax increases [22], there is a need to aggressively promote cessation services at the time of tobacco tax increases. Such promotions capitalize on the potential of tax increases to foster cessation before smokers adopt price-minimizing behaviors and continue to smoke.

Moreover, this study demonstrated the association of the state tax increase on Helpline enrollments and quitplan.com enrollments, controlling for media. To our knowledge, this has not been previously reported. The tax increase and media each explained about the same amount of the variance in Helpline enrollments (34% and 36.1%, respectively) and media explained 64% of the variance in quitplan.com enrollments. The combination of the tax increase and paid media explained over 70% of the variance in both Helpline and quitplan.com enrollments (70.1% and 71.6%, respectively). These findings suggest the need to carefully track paid media, earned media, and other environmental factors to learn what is most effective in driving tobacco users to cessation services and how to combine strategies to have additional impact. Further research is warranted to see whether the experience of other states with tax increases, media, and cessation service enrollments are similar to Minnesota’s.

Few changes in participant demographic, clinical and tobacco use characteristics were noted after the tax increase (see Tables 1 and 2). Our findings differ from those reported by Bush and Harwell [5,7]. Both of these studies found more variation in caller characteristics after tax increases than were found in this analysis. Looking only at the variables that were comparable across these studies and this analysis, Bush et al. [5] found that more callers after the federal tax increase were white, had less than a high school education, and smoked more cigarettes per day, while Harwell [7] found that callers after Montana’s tax increase were more likely to be white, female, younger, and smoked one or more packs of cigarettes per day. In contrast, both Helpline and quitplan.com enrollees were older. Additionally, more quitplan.com enrollees reported that they used cigarettes within 30 minutes of waking. It is unclear why few changes in enrollee characteristics were observed despite increased volumes and enrollments. These findings suggest that an array of communication channels and messages should be used to promote cessation services in order to reach as many diverse audiences as possible.

There are limitations to this study. First, data were available only from QUITPLAN Services, not from the quitlines run by major health plans in Minnesota or other cessation services offered in the state, so the overall association of the tax increase on cessation service utilization in Minnesota is underestimated. Second, other environmental factors that may have influenced smokers to seek cessation services during this time period were not controlled for; however, we were not aware of other significant shifts in the environment that may have accounted for these findings. Third, ClearWay Minnesota tags broadcast advertisements with quitplan.com more frequently than its telephone number (approximately 55%–60% of ads were tagged with quitplan.com during the 78-week period analyzed), which may partially account for broadcast media explaining more of the variation in quitplan.com enrollments compared to the Helpline. It is important to note that all ads that were broadcast during the six weeks around the tax increase were tagged equally with the QUITPLAN Helpline telephone number and quitplan.com. Fourth, ClearWay Minnesota has a year-round media presence promoting QUITPLAN Services, so there was not an opportunity to measure the association of the tax increase without media. Finally, cessation outcomes were not measured in this study.

This analysis identifies several areas for further research. How to optimally leverage tax increases and sustain the interest in and use of quitlines and web-based cessation programs over more than a four to six-week time period is unknown. Research is needed on innovative strategies to maximize the impact of tax increases on both volumes and enrollments in cessation services. Additionally, there is a need to better understand how to effectively engage all types of smokers in the quitting process. This may require carefully examining current cessation service offerings, using technologies such as text messaging to reach different populations of smokers, and partnering with organizations serving diverse communities and persons of low socioeconomic status to facilitate linkages to cessation services. This is essential in order to reach the United States goal in Healthy People 2020 (www.healthypeople.gov/2020/default.aspx) of 80% of smokers making quit attempts.

## Conclusions

Minnesota’s tobacco tax increase and concurrent media campaign was associated with substantial increases in quitline call volumes, web visits, and enrollments in cessation services. Cigarette tax increases are powerful policy changes, and the evidence supporting their association with changing tobacco use patterns is clear. In most states, they occur infrequently. When tax increases do occur, it is incumbent on the tobacco control, public health, and healthcare communities to leverage the power of tax increases and concurrent media campaigns to increase quit attempts, cessation service utilization, and ultimately long-term cessation. Our window of opportunity is narrow, and we must seize it.
